# Perforated Meckel’s diverticulitis secondary to a chicken bone mimicking acute appendicitis: a case report

**DOI:** 10.1097/MS9.0000000000005804

**Published:** 2026-10-22

**Authors:** Musab Basem Dwaik, Bayan Basem Alshaer, Yazan Jawad Janazreh, Ibrahim Mohammad Alkhamaise, Majd Zoghayyer, Abdullah Ibraheem Bregheith, Moayad Alameh, Mohammad Jamil Alhashlamoun

**Affiliations:** a Faculty of Medicine, Al-Quds University, Jerusalem, Palestine; b Surgery Department, Al-Ahli Hospital, Hebron, Palestine

**Keywords:** acute abdomen, acute appendicitis mimic, foreign body, laparoscopy, Meckel’s diverticulitis, Meckel’s diverticulum

## Abstract

**Background:**

Meckel’s diverticulum is the most common congenital anomaly of the gastrointestinal tract. Symptomatic presentation in adults is uncommon and often mimics acute appendicitis, posing a diagnostic challenge.

**Case Presentation:**

We report a 49-year-old man who presented with right lower quadrant abdominal pain suggestive of acute appendicitis. Ultrasound demonstrated a non-compressible tubular structure in the right iliac fossa. Contrast-enhanced computed tomography (CT) revealed an inflamed Meckel’s diverticulum with a linear hyperdense foreign body extending into the diverticular wall. The appendix appeared normal. The patient underwent laparoscopic diverticulectomy, which confirmed a concealed perforation caused by a chicken bone. Histopathology revealed ectopic pancreatic tissue with chronic active inflammation. The postoperative course was uneventful.

**Conclusion:**

This case highlights the diagnostic challenge of Meckel’s diverticulitis in adults and emphasizes the importance of CT imaging and histopathological evaluation, particularly in rare cases involving foreign body impaction.

## Introduction

Meckel’s diverticulum (MD) is the most common congenital gastrointestinal anomaly, resulting from incomplete obliteration of the vitelline duct during the weeks 5–7 of embryological development^[^1,2^]^. Affecting approximately 2% of the population, it follows the “rule of twos”: it is located nearly 2 inches from the ileocecal valve, measures about 2 inches in length, and is more frequently identified in males^[^3,4^]^. While often asymptomatic, MD can present with complications such as intestinal obstruction, inflammation, or perforation, often due to acid secretion from ectopic gastric mucosa^[^1,4,5^]^.

Meckel’s diverticulitis is a serious complication that can progress to perforation. Clinically, it often mimics acute appendicitis, making preoperative diagnosis exceptionally difficult^[^1,6,7^]^. In equivocal cases, laparoscopy is a valuable diagnostic and therapeutic tool^[^7,8^]^. Foreign body (FB) ingestion is a rare cause of MD perforation. While most ingested objects pass uneventfully, sharp materials such as chicken or fish bones can cause localized pressure necrosis and subsequent perforation^[^5,9^]^. Despite being a rare complication, identified in only 5–8% of surgically managed cases, it represents a surgical emergency^[^5,9^]^.

HIGHLIGHTS

- Meckel’s diverticulitis in adults can clinically and radiologically mimic acute appendicitis.
- Contrast-enhanced computed tomography is crucial in identifying intraluminal foreign bodies that cause perforation.
- Ingested chicken bones are a rare but serious cause of perforation of Meckel’s diverticulum.
- Ectopic pancreatic tissue may play a role in the pathogenesis of diverticulitis.
- Laparoscopic diverticulectomy is a safe and effective treatment for complicated Meckel’s diverticulum.

This case report, prepared in accordance with the CARE guidelines, describes a rare presentation of Meckel’s diverticulitis and perforation caused by an ingested chicken bone, mimicking acute appendicitis in an adult patient.

This case report is presented in accordance with the CARE reporting guidelines. The authors have completed the CARE checklist for case reports, which is included as a Supplemental Digital Content File, available at: https://links.lww.com/MS9/B469.

## Case presentation

A 49-year-old male patient of Palestinian ethnicity presented to the emergency department with a 2-day history of acute abdominal pain. The pain was initially a diffuse discomfort, more prominent in the left upper quadrant, before migrating to the right iliac fossa on the day of admission. The pain was colicky, severe in intensity, and progressive, with a transient decrease followed by exacerbation.

The pain was associated with a feverish sensation, watery diarrhea, nausea, and anorexia. There was no history of vomiting, urinary symptoms, chest pain, constipation, weight loss, or rectal bleeding. The patient denied any prior similar episodes.

His past medical history was unremarkable. Surgical history was significant for a laparoscopic cholecystectomy performed 2 years ago. He was not taking any regular medications and had no known drug allergies.

On examination, the patient appeared well and was not in acute distress. His vital signs were within normal limits. Abdominal examination revealed a soft, non-distended abdomen with localized tenderness in the right iliac fossa, associated with guarding and rebound tenderness. Rovsing’s sign was positive, while no rigidity was noted. The remainder of the systemic examination was unremarkable.

Initial laboratory and imaging findings suggested an inflammatory intra-abdominal process. Detailed laboratory and radiological findings are described in the Investigations section.

Given the imaging findings and the risk of complications, surgical intervention was planned following initial stabilization.

## Investigations

### Laboratory findings

Initial laboratory investigations obtained upon admission revealed a white blood cell count of 9.8 × 10^9^/L with relative neutrophilia (72.2%). Hemoglobin was 15.2 g/dL, and platelet count was 234 × 10^9^/L. Renal function tests were within normal limits, with a creatinine level of 1.19 mg/dL and a blood urea nitrogen of 13.4 mg/dL.

Follow-up inflammatory markers on hospital day 4 demonstrated an elevated C-reactive protein level of 15.1 mg/L (normal reference range: <10 mg/L), indicating an ongoing inflammatory process.

### Ultrasound findings

An abdominal ultrasound performed prior to computed tomography (CT) imaging demonstrated a diffuse inflammatory process in the right iliac fossa surrounding a tubular, non-compressible structure measuring approximately 1.8 cm in maximal diameter. These findings were suggestive of complicated appendicitis versus a perforated ileal segment.

### CT findings

A contrast-enhanced CT scan of the abdomen and pelvis revealed a blind-ending tubular outpouching arising from the distal ileum along the antimesenteric border, measuring approximately 3 cm in diameter. The lesion demonstrated marked wall thickening with submucosal edema (approximately 1.2 cm) and surrounding mesenteric fat stranding (Fig. 1).

**Figure 1.**
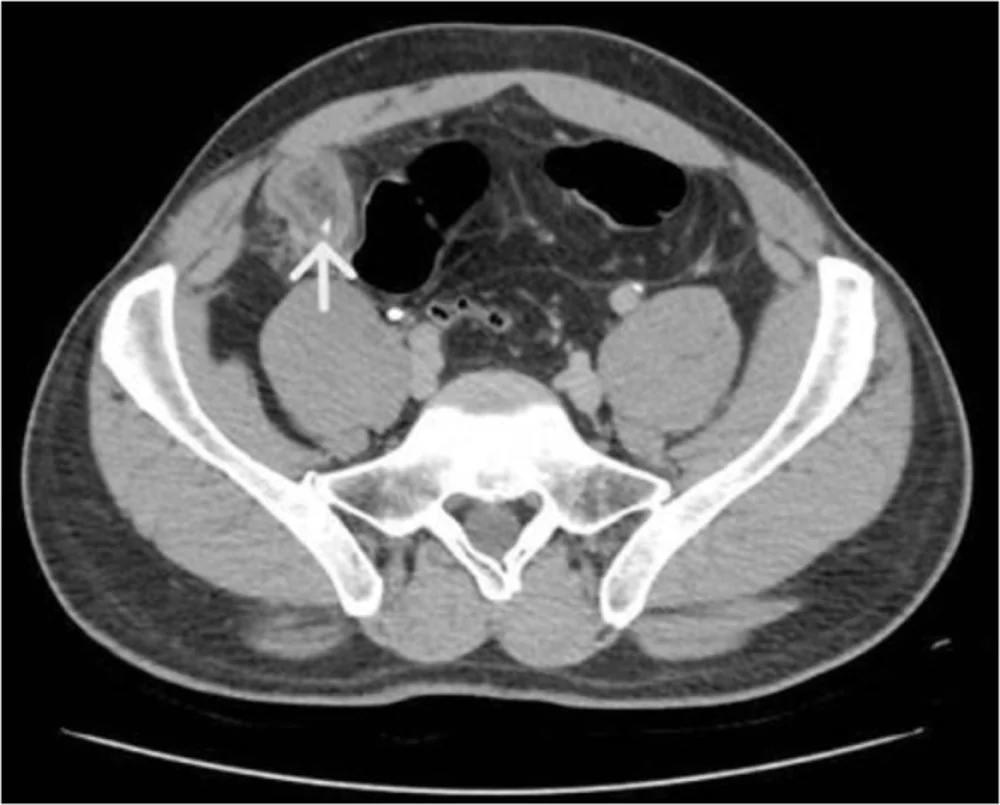
Contrast-enhanced CT scan (axial view) demonstrating an inflamed Meckel’s diverticulum (arrow) with an intraluminal, linear, hyperdense foreign body that extends into the diverticular wall and is accompanied by surrounding fat stranding.

A linear hyperdense structure measuring approximately 1.5 cm was identified within the lumen, extending into the diverticular wall and suggestive of an intraluminal FB (likely a chicken bone) (Fig. 1).

The appendix appeared normal in caliber, gas-filled, with no surrounding inflammatory changes. No evidence of perforation, abscess formation, or bowel obstruction was identified. Mild reactive mucosal thickening of the cecum was also noted.

These findings were consistent with Meckel’s diverticulitis.

### Diagnostic challenge (why it mimicked appendicitis)

The initial clinical presentation and ultrasound findings were highly suggestive of acute appendicitis based on right lower quadrant pain and a non-compressible tubular structure in the right iliac fossa. However, CT imaging demonstrated a normal appendix and identified an inflamed MD as the underlying pathology, highlighting the diagnostic challenge and the importance of cross-sectional imaging in atypical presentations.

### Management

Upon admission, the initial approach to management was conservative and consisted of intravenous fluid resuscitation, bowel rest, analgesia, and intravenous antibiotics. The patient showed partial clinical improvement and remained hemodynamically stable. Nevertheless, the presence of a retained sharp FB (specifically, a chicken bone) extending into the diverticular wall posed a significant risk of progression to perforation and other complications. Therefore, surgical intervention was deemed necessary.

On hospital day 3, the patient underwent laparoscopic diverticulectomy under general anesthesia. Intraoperative exploration revealed Meckel’s diverticulitis complicated by a concealed perforation at the site of an impacted FB (specifically, a chicken bone), with the defect walled off by omental adhesions. No free pus, peritoneal collection, or generalized peritonitis was identified. The appendix was visualized and confirmed to be grossly normal (Fig. 2).

**Figure 2.**
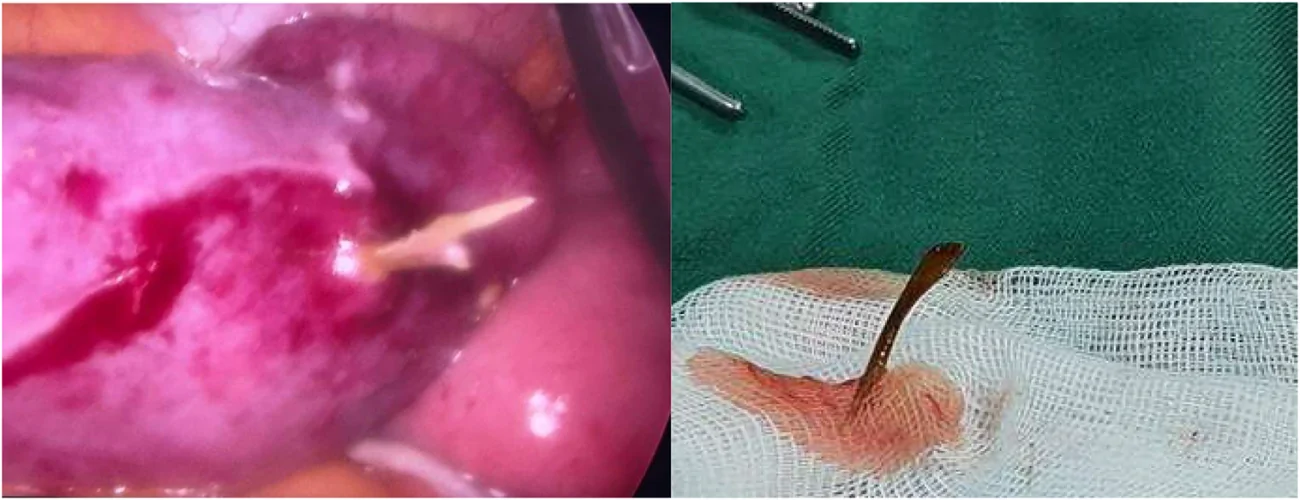
Left: An intraoperative image showing Meckel’s diverticulitis complicated by a chicken bone-induced concealed perforation with omental adhesion and no pus or collection. Right: The extracted foreign body, which was confirmed to be a chicken bone.

Evaluation of the diverticular base demonstrated a relatively wide and well-demarcated neck without extensive base necrosis, ischemia of the adjacent antimesenteric ileal border, or compromise of the surrounding small-bowel lumen. Given the absence of a narrow or indurated base and no evidence of multifocal diverticular pathology, diverticulectomy was chosen over a segmental small-bowel resection to successfully remove the pathology and the FB while preserving optimal intestinal length. The diverticulum was successfully resected using an endoscopic stapling device, and the specimen was retrieved without complications (Fig. 2).

### Postoperative follow-up

The patient had an uneventful postoperative recovery and was discharged on postoperative day 4. At the 2-week and 1-month outpatient follow-up visits, the patient remained asymptomatic, with complete wound healing and no evidence of abdominal complications or recurrence of symptoms.

### Histopathology

Histopathological examination of the resected specimen confirmed an MD measuring approximately 2 cm. Ectopic pancreatic tissue was identified within the diverticulum. Microscopic examination demonstrated chronic active inflammation with prominent granulation tissue formation.

No evidence of dysplasia or malignancy was identified.

### Patient perspective

Following the successful laparoscopic procedure and smooth recovery, the patient expressed profound relief and gratitude for the timely diagnosis and intervention. He noted that the explanation provided by the medical team regarding his condition, specifically, the discovery of the FB within the anomaly, helped him fully understand the cause of his sudden illness, and he reported high satisfaction with the overall hospital course and post-discharge recovery.

## Discussion

### Overview of MD

MD is the most common congenital anomaly of the gastrointestinal tract, resulting from incomplete obliteration of the omphalomesenteric (vitelline) duct during embryologic development. It is a true diverticulum containing all layers of the intestinal wall and is typically located on the antimesenteric border of the ileum, approximately 60–100 cm proximal to the ileocecal valve. Although present in about 2% of the population, only a small proportion (4–6%) become symptomatic during their lifetime, usually due to complications such as bleeding, inflammation, obstruction, or perforation related to ectopic gastric or pancreatic mucosa^[^1,10,11^]^.

### Adult vs pediatric presentation

The clinical presentation of MD differs significantly between pediatric and adult populations. In children, the most common manifestation is painless lower gastrointestinal bleeding due to acid secretion from ectopic gastric mucosa, which causes ulceration of the adjacent ileal mucosa. Other pediatric presentations include intussusception and bowel obstruction. In contrast, adults more commonly present with complications such as diverticulitis, perforation, or intestinal obstruction. Importantly, symptomatic MD in adults is often misdiagnosed as acute appendicitis or other causes of an acute abdomen due to overlapping clinical features and low preoperative suspicion, leading to delayed or incidental diagnosis^[^1,10,11^]^.

### Mimicking appendicitis (diagnostic challenge)

Meckel’s diverticulitis frequently mimics acute appendicitis, particularly in adult patients. Patients typically present with right lower quadrant abdominal pain, fever, and signs of peritoneal irritation, which are clinically indistinguishable from appendicitis. As a result, preoperative diagnosis is rarely achieved, and many cases are identified intraoperatively during exploration for suspected appendicitis when the appendix appears normal. This highlights the diagnostic challenge and emphasizes that MD should be considered in the differential diagnosis of acute abdomen, especially when imaging or intraoperative findings are inconclusive^[^1,10^]^.

### FB-induced Meckel’s diverticulitis

FB-induced inflammation of MD is a rare but well-documented cause of diverticulitis and perforation. Reported foreign bodies include fish bones, chicken bones, toothpicks, and other indigestible materials that may become lodged within the diverticulum, leading to luminal obstruction, mucosal injury, and subsequent inflammation or perforation. The blind-ended structure and relatively narrow lumen of the diverticulum predispose it to the retention of such materials. In most reported cases, diagnosis is established intraoperatively due to nonspecific clinical presentation and limited preoperative suspicion. However, the identification of a linear hyperdense structure on CT, as demonstrated in our case, can serve as an important diagnostic clue suggesting an FB etiology^[^12–14^]^.

Ectopic tissue is frequently identified in MD, with gastric and pancreatic tissues being the most commonly reported types. The presence of ectopic mucosa may contribute to the development of complications, including inflammation, ulceration, and perforation. In particular, ectopic pancreatic tissue, although less common than gastric mucosa, has been associated with chronic inflammatory changes and may play a role in the pathogenesis of diverticulitis. In the present case, histopathological examination confirmed the presence of ectopic pancreatic tissue, which may have contributed to the inflammatory process in addition to the mechanical effect of the impacted FB^[^1,15^]^.

### CT imaging vs surgical diagnosis

CT plays a central role in the evaluation of patients presenting with acute abdomen and may aid in the preoperative diagnosis of Meckel’s diverticulitis. Typical CT findings include a blind-ending tubular structure arising from the distal ileum, mural thickening, and surrounding mesenteric fat stranding. Despite these characteristic features, the sensitivity of CT remains limited, particularly in uncomplicated cases or when the diverticulum is misinterpreted as a small-bowel loop or the appendix. As a result, definitive diagnosis is frequently established intraoperatively, especially in patients undergoing surgery for suspected acute appendicitis with a normal appendix. This underscores the importance of maintaining a high index of suspicion, even in the era of advanced imaging modalities^[^1,12,16^]^.

### Management and surgical considerations

Surgical resection remains the definitive treatment for symptomatic MD. The choice between diverticulectomy and segmental ileal resection depends on intraoperative findings, including the degree of inflammation, involvement of the base, presence of perforation, and suspicion of ectopic tissue. Minimally invasive approaches, particularly laparoscopy, are increasingly favored due to reduced postoperative morbidity and faster recovery. However, controversy persists regarding the management of incidentally discovered MD, with no clear consensus on whether prophylactic resection should be routinely performed. In symptomatic cases, particularly those complicated by inflammation or FB impaction, prompt surgical intervention is recommended to prevent progression to perforation or abscess formation^[^10,11,16^]^.

### Literature comparison

Several studies have emphasized the rarity of symptomatic MD in older adults and the diagnostic challenges associated with its presentation. Cases involving FB impaction are particularly uncommon and are typically reported as isolated case reports. Similar to our case, previously published reports have described patients presenting with an acute abdomen mimicking appendicitis, with the diagnosis ultimately established intraoperatively or through CT imaging. Our case contributes to the existing literature by demonstrating clear radiological identification of a FB within an inflamed MD, highlighting the value of CT in facilitating preoperative diagnosis and guiding surgical management^[^12–15^]^.

### Limitations of the study

This case report has certain limitations that should be acknowledged. Primarily, as a single case report, our findings reflect the experience of one institution and may not be generalizable to all clinical settings. Additionally, while the identification of an FB within the MD was successful by CT imaging, preoperative diagnosis of MD diverticulitis remains challenging, often relying on high-resolution imaging and a high index of clinical suspicion. Finally, the retrospective nature of our data collection may limit the depth of longitudinal follow-up observations. Nonetheless, this report contributes to the existing literature by highlighting the diagnostic value of cross-sectional imaging in patients presenting with an acute abdomen.

## Conclusion

Meckel’s diverticulitis should be considered in adult patients presenting with an acute abdomen, particularly when imaging findings are atypical or when the appendix appears normal. FB impaction and ectopic tissue are important contributors to complications and require timely surgical intervention.

## Ethical approval

Ethical approval was not required for this case report in accordance with the applicable institutional policy.

## Data Availability

All data related to this case report are included within the manuscript and its accompanying figures. Any further inquiries can be directed to the corresponding author.

## Research registration unique identifying number (UIN)

Not applicable. This is a case report and does not involve a clinical trial or a new surgical technique that requires registration in a public database.
